# A Multiagent Alphavirus DNA Vaccine Delivered by Intramuscular Electroporation Elicits Robust and Durable Virus-Specific Immune Responses in Mice and Rabbits and Completely Protects Mice against Lethal Venezuelan, Western, and Eastern Equine Encephalitis Virus Aerosol Challenges

**DOI:** 10.1155/2018/8521060

**Published:** 2018-06-03

**Authors:** Lesley C. Dupuy, Michelle J. Richards, Brian D. Livingston, Drew Hannaman, Connie S. Schmaljohn

**Affiliations:** ^1^United States Army Medical Research Institute of Infectious Diseases, Fort Detrick, Frederick, MD 21702-5011, USA; ^2^Ichor Medical Systems, Inc., San Diego, CA 92121-3209, USA

## Abstract

There remains a need for vaccines that can safely and effectively protect against the biological threat agents Venezuelan (VEEV), western (WEEV), and eastern (EEEV) equine encephalitis virus. Previously, we demonstrated that a VEEV DNA vaccine that was optimized for increased antigen expression and delivered by intramuscular (IM) electroporation (EP) elicited robust and durable virus-specific antibody responses in multiple animal species and provided complete protection against VEEV aerosol challenge in mice and nonhuman primates. Here, we performed a comparative evaluation of the immunogenicity and protective efficacy of individual optimized VEEV, WEEV, and EEEV DNA vaccines with that of a 1 : 1 : 1 mixture of these vaccines, which we have termed the 3-EEV DNA vaccine, when delivered by IM EP. The individual DNA vaccines and the 3-EEV DNA vaccine elicited robust and durable virus-specific antibody responses in mice and rabbits and completely protected mice from homologous VEEV, WEEV, and EEEV aerosol challenges. Taken together, the results from these studies demonstrate that the individual VEEV, WEEV, and EEEV DNA vaccines and the 3-EEV DNA vaccine delivered by IM EP provide an effective means of eliciting protection against lethal encephalitic alphavirus infections in a murine model and represent viable next-generation vaccine candidates that warrant further development.

## 1. Introduction

Venezuelan equine encephalitis virus (VEEV), western equine encephalitis virus (WEEV), and eastern equine encephalitis virus (EEEV) are nonsegmented, positive-sense RNA viruses of the genus *Alphavirus* in the family *Togaviridae* [1]. Naturally transmitted by mosquitoes through rodent or bird hosts, VEEV, WEEV, and EEEV are highly pathogenic for equines and humans and have caused periodic epizootics throughout North, Central, and South America [2]. Human infection with these New World alphaviruses typically results in an acute, incapacitating disease characterized by fever, headache, nausea, myalgia, and malaise [3]. Severe neurological disease, including fatal encephalitis, can also result from VEEV, WEEV, and EEEV infection of humans. Although the human case-fatality rates associated with natural infection are estimated to be low for VEEV (≤1%) and intermediate for WEEV (3–15%), EEEV is the most severe of the arboviral encephalitides with a human case-fatality rate estimated to be from 33% to as high as 75% [4–7]. Moreover, numerous documented laboratory accidents and the results of animal studies have demonstrated that VEEV, WEEV, and EEEV are also highly infectious in aerosols, and infection with aerosolized virus could potentially result in higher human mortality than that observed with natural infection [8–10]. In addition to producing incapacitating or lethal infections and being infectious in aerosols, these encephalitic alphaviruses are also easily grown to high titers in inexpensive and unsophisticated cell culture systems and are considerably stable [4]. Consequently, VEEV, WEEV, and EEEV represent significant biological defense threats and are classified as Category B priority pathogens by both the Centers for Disease Control and Prevention and the National Institute of Allergy and Infectious Diseases.

Although there are no licensed human vaccines for the encephalitic alphaviruses, live-attenuated and formalin-inactivated vaccines are currently utilized under US Food and Drug Administration Investigational New Drug (IND) status to protect laboratory workers and other at-risk personnel. The live-attenuated VEEV IND vaccine, TC-83, provides long-lasting immunity and protection from both subcutaneous and aerosol VEEV challenges; however, it causes significant adverse reactions in approximately 25% of recipients, and approximately 20% of recipients fail to develop a detectable neutralizing antibody response [11, 12]. The formalin-inactivated VEEV IND vaccine derived from TC-83, C-84, and the formalin-inactivated WEEV and EEEV IND vaccines are well tolerated, but they require frequent boosting to elicit and maintain detectable neutralizing antibody responses in humans and have exhibited suboptimal protection against aerosol viral challenge in animal studies [13–15]. In addition, immune interference has been documented when the VEEV, WEEV, and EEEV IND vaccines are administered simultaneously or sequentially in humans [16–18]. Due to the significant limitations associated with these existing vaccine candidates, they are not being pursued for licensure. As a result, development of improved vaccines that can safely and effectively protect humans against encephalitic alphavirus infections is needed [19]. Toward this goal, next-generation encephalitic alphavirus vaccine candidates, including live-attenuated, inactivated, Sindbis virus-based chimeric, virus replicon particle, virus-like particle, DNA, and virus-vectored vaccines, are all currently at various stages of development [20–22].

Vaccination with DNA plasmids that express protein antigens has numerous inherent advantages as a platform for the development of next-generation vaccines. Foremost among the benefits of this approach is that the endogenous expression of target antigens achieved with DNA vaccination can elicit both cellular and humoral immune responses [23–26]. Due to the lack of a host immune response to the vector backbone, DNA vaccines also circumvent issues of preexisting or vaccine-induced vector-based immunity that can deleteriously affect vaccine immunogenicity and safety [27, 28]. From a logistical standpoint, DNA vaccines can be rapidly developed and produced using well-established manufacturing procedures and without the need to propagate a pathogen or inactivate an infectious organism. DNA vaccines can also be readily formulated to generate multiagent vaccines [29]. Importantly, DNA vaccines have also exhibited a favorable safety profile in numerous human clinical trials [30]. Despite these promising characteristics, the primary limitation of this approach has been suboptimal immunogenicity in humans when administered by conventional injection. To address this, we have pursued a range of strategies for enhancing the potency of encephalitic alphavirus DNA vaccines to include investigation of alternative delivery methods and refinement of the coding sequences for the target antigens.

In our previous studies, a DNA vaccine expressing the structural proteins (C-E3-E2-6K-E1) of VEEV subtype IAB (strain Trinidad donkey) from the wild-type genes administered by particle-mediated epidermal delivery (PMED) or “gene gun” elicited strong virus-specific antibody responses in multiple animal species; however, the virus-neutralizing antibody responses were low and only partial protection against homologous VEEV aerosol challenge was observed in mice and nonhuman primates (NHPs) [31–33]. We subsequently employed directed molecular evolution or “gene shuffling” of VEEV, WEEV, and EEEV envelope glycoprotein genes in an attempt to improve the neutralizing antibody response to VEEV, WEEV, and EEEV DNA vaccines. Although DNA vaccines expressing certain variant envelope glycoproteins elicited increased VEEV IAB-neutralizing antibody titers compared to the wild-type parental VEEV DNA vaccine and provided improved protection against VEEV IAB aerosol challenge in mice when delivered by PMED, these studies failed to identify variant envelope glycoprotein DNA vaccines exhibiting increased immunogenicity against WEEV and EEEV as compared to the wild-type parental WEEV and EEEV DNA vaccines [32]. More recently, we optimized the VEEV DNA vaccine for increased mammalian expression of the structural proteins by adapting the gene sequence to reflect the codon bias of highly expressed *Homo sapiens* genes, adjusting regions of very high (>80%) or very low (<30%) guanine-cytosine content, and avoiding *cis*-acting motifs that can negatively impact mRNA expression or stability. Because earlier studies by others indicated that the capsid protein of VEEV and EEEV can be cytotoxic and can inhibit cellular transcription and nuclear import and export in vertebrate cells [34–37], we also eliminated the capsid gene from this construct. When delivered by intramuscular (IM) electroporation (EP), the optimized VEEV DNA vaccine elicited significantly improved virus-specific antibody responses, including increased levels of virus-neutralizing antibodies, in multiple animal species and provided complete protective immunity against homologous VEEV aerosol challenge in mice and NHPs [38]. Subsequently, this VEEV DNA vaccine candidate delivered by IM or intradermal (ID) EP proved to be safe, tolerable, and immunogenic in humans in a recently completed Phase 1 clinical trial [39].

The primary objective of the studies reported here was to apply this approach in an attempt to develop fully protective DNA vaccines for WEEV and EEEV. However, our ultimate goal is to develop a single multiagent vaccine formulation capable of eliciting protective immunity against VEEV, WEEV, and EEEV. Therefore, we performed a comparative evaluation of the immunogenicity and protective efficacy of the individual optimized VEEV, WEEV, and EEEV DNA vaccines with that of a 1 : 1 : 1 mixture of these vaccines, which we have termed the 3-EEV DNA vaccine, when delivered by IM EP in mice. To directly compare the results obtained for the DNA vaccines with those achieved with the vaccines currently used to protect at-risk personnel, mice vaccinated with the live-attenuated VEEV IND vaccine TC-83 or the formalin-inactivated WEEV or EEEV IND vaccines were also included in these studies. We also assessed the virus-neutralizing antibody responses elicited by the individual VEEV, WEEV, and EEEV and 3-EEV DNA vaccines delivered by IM EP in rabbits.

## 2. Materials and Methods

### 2.1. Ethics Statement

All animal research was conducted in compliance with the Animal Welfare Act and other federal statutes and regulations relating to animals and experiments involving animals and adheres to principles stated in the “Guide for the Care and Use of Laboratory Animals,” Institute for Laboratory Animal Research, Division of Earth and Life Studies, National Research Council, National Academies Press, Washington, DC, 2011. The United States Army Medical Research Institute of Infectious Diseases (USAMRIID) facility where this animal research was conducted is fully accredited by the Association for the Assessment and Accreditation of Laboratory Animal Care International.

### 2.2. Vaccines

Codon-optimized WEEV and EEEV structural genes were generated by subjecting the wild-type 26S structural gene sequences minus the capsid protein coding region (E3-E2-6K-E1) of WEEV strain CBA87 (GenBank accession number DQ432026) and EEEV strain FL91-4679 (GenBank accession number AY705241) to the GeneOptimizer™ bioinformatic algorithm for optimized expression in *Homo sapiens* followed by synthesis of the codon-optimized genes (Geneart, Regensburg, Germany) as done previously for VEEV IAB strain Trinidad donkey (GenBank accession number L01442) [38]. As done previously for VEEV [38], WEEV and EEEV DNA vaccine plasmids were then constructed by inserting the synthesized codon-optimized genes into the *Not*I and *Bgl*II restriction sites of the eukaryotic expression vector pWRG7077 (PowderJect, Madison, WI), which has been described previously [40]. Endotoxin-free, research-grade plasmids used in these studies were manufactured by Aldevron (Fargo, ND). The live-attenuated VEEV vaccine TC-83 (NDBR 102, Lot 4 Run 3) used in these studies was manufactured by the National Drug Company (Swiftwater, PA). The inactivated WEEV (TSI-GSD-210, Lot 2-1-91) and EEEV (TSI-GSD-104, Lot 2-1-89) vaccines used in these studies were manufactured by the Government Services Division of the Salk Institute (Swiftwater, PA).

### 2.3. Animals, Vaccinations, and Blood Collections

Female BALB/c mice (6–8 weeks old, Charles River Laboratories, Wilmington, MA) and New Zealand White rabbits (3–3.5 kg, Charles River Laboratories) were vaccinated with plasmid DNA diluted to the appropriate concentration as described in the text and shown in the figures in calcium- and magnesium-free phosphate-buffered saline (Invitrogen, Carlsbad, CA) by IM EP using the TriGrid™ Delivery System (Ichor Medical Systems, San Diego, CA) as described previously [41]. Briefly, mice anesthetized with IM injection of a diluted acepromazine/ketamine/xylazine mixture or with isoflurane gas were injected into one tibialis anterior muscle with 20 *μ*l of DNA solution using a 3/10 ml U-100 insulin syringe (Becton-Dickinson, Franklin Lakes, NJ) inserted into the center of a TriGrid electrode array with 2.5 mm electrode spacing. Rabbits anesthetized with isoflurane gas were injected into one quadriceps muscle with 0.5 ml of DNA solution using a 1 ml syringe (Becton-Dickinson) inserted into the center of a TriGrid electrode array with 6.0 mm electrode spacing. Injection of DNA was followed immediately by electrical stimulation at amplitude of 250 V/cm, and the total duration was 40 ms over a 400 ms interval. The live-attenuated VEEV vaccine TC-83 and inactivated WEEV and EEEV vaccines were delivered to mice as 0.5 ml doses by subcutaneous injection. At various times after vaccination as described in the text and shown in the figures, blood samples were collected from anesthetized mice by retroorbital or submandibular vein bleed and from anesthetized rabbits by central auricular artery bleed, and serum was recovered by centrifugation.

### 2.4. ELISA Assays

Total IgG anti-VEEV, WEEV, or EEEV antibody titers were determined for serum samples by indirect enzyme-linked immunosorbent assay (ELISA) using sucrose-purified, irradiated whole VEEV IAB strain Trinidad donkey, WEEV strain CBA87, or EEEV strain FL91-4679 antigen as described previously [42]. Briefly, twofold serial dilutions of sera starting at 1 : 100 were incubated with 250 ng per well of antigen in 96-well plates (Corning, Corning, NY). Horseradish peroxidase- (HRP-) conjugated anti-mouse IgG antibodies (Sigma-Aldrich, St. Louis, MO) and ABTS peroxidase substrate (KPL, Gaithersburg, MD) were used for detection. The optical density at 405 nm was determined using a SpectraMax M2e microplate reader (Molecular Devices, Sunnyvale, CA), and the endpoint titers were calculated in SoftMax Pro v5 (Molecular Devices) using a 4-parameter logistic curve fit and a cutoff value equal to the mean optical density of the negative control samples plus three standard deviations.

### 2.5. PRNT Assays

Virus-neutralizing antibody titers against VEEV subtypes IAB (strain Trinidad donkey), IC (strain 6119), ID (strain 3880), and IE (strain 68U201) as well as Mucambo virus (MUCV, formerly VEEV subtype IIIA, strain BeAn8), WEEV (strain CBA87), and EEEV (strain FL91-4679) were determined for serum samples by the plaque reduction neutralization test (PRNT) as described previously [42]. Briefly, twofold serial dilutions of sera starting at 1 : 20 were mixed with equal volumes of medium containing ~200 PFU of virus and incubated for 24 h at 4°C. The virus/antibody mixtures were then used to infect confluent monolayers of Vero cells contained in six-well plates (Corning) for 1 h at 37°C after which an overlay consisting of 0.6% agar (GeneMate, Kaysville, UT) in complete Eagle's basal medium with Earle's salts (EBME) without phenol red (Invitrogen) was added. The plates were stained 24 h later by the addition of an overlay containing 5% neutral red (Gibco, Gaithersburg, MD) and 0.6% agar in complete EBME without phenol red, and the plaques were counted 24 h after staining. The neutralizing antibody titers were then calculated as a reciprocal of the highest dilution resulting in an 80% reduction of the plaque number as compared to virus-only control wells.

### 2.6. ELISpot Assays

Anti-VEEV cellular immune responses were analyzed by interferon- (IFN-) *γ* enzyme-linked immunospot (ELISpot) assay using standard methods as described previously [43]. Briefly, splenocytes isolated from individual spleens obtained from vaccinated mice using 100 *μ*M nylon cell strainers (Corning) were resuspended in complete RPMI 1640 medium (Mediatech, Manassas, VA). The resuspended splenocytes from each spleen were then added at a concentration of 2 × 10^5^ cells per well to triplicate wells of MultiScreen_HTS_ IP 0.45 *μ*m PVDF filter 96-well plates (Millipore, Billerica, MA) previously coated with mouse IFN-*γ* ELISpot capture antibody (Becton-Dickinson). The splenocytes were then cultured with no peptide, 10 *μ*g/ml of concanavalin A (Sigma-Aldrich), 20 *μ*g/ml of *β*-galactosidase peptide TPHPARIGL (New England Peptide, Gardner, MA), or 10 *μ*g/ml of pooled 15-mer peptides with an 11-base overlap spanning the VEEV IAB E2 or E1 envelope glycoprotein (Pepscan, Lelystad, Netherlands) for 24 h at 37°C with 5% CO_2_. Secreted IFN-*γ* was detected by aspirating the cell suspension and successively incubating the plate for 2 h at room temperature with mouse IFN-*γ* ELISpot detection antibody (Becton-Dickinson), for 1 h at room temperature with streptavidin-HRP (Becton-Dickinson), and for 20 min at room temperature with 3-amino-9-ethylcarbazole (AEC) substrate (Becton-Dickinson). The substrate reaction was then stopped by washing the plates with deionized H_2_O, the plates were dried for 2 h at room temperature, and the spots were enumerated.

### 2.7. Aerosol Challenge of Mice

Mice were placed into a class III biological safety cabinet located inside a biosafety level 3 containment suite and exposed in a whole-body aerosol chamber to a VEEV, WEEV, or EEEV aerosol created by a Collison nebulizer for 10 min as previously described [44]. Sucrose-purified VEEV IAB strain Trinidad donkey, WEEV strain CBA87, or EEEV strain FL91-4679 was diluted to an appropriate starting concentration in Hank's Balanced Salt Solution (Gibco) containing 1% fetal bovine serum (Thermo Fisher Scientific, Waltham, MA) for use in aerosol generation. Samples collected from the all-glass impinger attached to the aerosol chamber were analyzed by plaque assay on Vero cells using standard methods as previously described to determine the inhaled dose of VEEV, WEEV, or EEEV [45]. The mice were monitored at least twice daily for clinical signs of disease and survival for 28 days postchallenge, and any animals found to meet early endpoint criteria were euthanized.

### 2.8. Statistical Methods

Log_10_ transformations were applied to whole-virus ELISA titers and PRNT_80_ titers for analyses. Mixed model analysis of variance (ANOVA) with post hoc Tukey's tests was used for pairwise comparisons of ELISA and PRNT_80_ titers and ELISpot counts with the same stimulation condition between groups at each time point. Paired *t*-tests were used to compare ELISA and PRNT_80_ titers and ELISpot counts for different stimulation conditions within groups. Kaplan-Meier survival analysis and log-rank tests with stepdown Sidak adjustment was used for comparison of survival curves between groups. Fisher's exact tests with stepdown bootstrap adjustment were used to compare survival rates between groups. *t*-tests with stepdown bootstrap adjustment were used to compare mean times-to-death between groups. The effects of ELISA and PRNT_80_ titers on the probability of survival were assessed using a backwards-selection logistic regression model. Analyses were conducted using SAS v9.2 (SAS Institute, Cary, NC). Statistical significance was defined as *p* < 0.05 in all tests.

## 3. Results

### 3.1. VEEV-Specific Antibody Responses of Vaccinated Mice

To first compare the immunogenicity and protective efficacy of the individual optimized VEEV DNA vaccine to that of a 1 : 1 : 1 mixture of the optimized VEEV, WEEV, and EEEV DNA vaccines (3-EEV DNA vaccine), female BALB/c mice (*n* = 10 per group) were vaccinated on days 0 and 21 with 5 *μ*g of the VEEV plasmid or with 5 *μ*g of each of the VEEV, WEEV, and EEEV plasmids (15 *μ*g total) by IM EP. Negative control mice (*n* = 10) were vaccinated on days 0 and 21 with 5 *μ*g of the empty vector plasmid by IM EP. To allow comparison to the live-attenuated VEEV IND vaccine, mice (*n* = 10) received a single administration of the human dose of 0.5 ml of TC-83 (1 × 10^4^ PFU) by subcutaneous injection on day 0. Serum samples obtained on days 21 and 42 were assayed for total IgG anti-VEEV antibodies by ELISA and for VEEV-neutralizing antibodies by PRNT.

**Figure 1:**
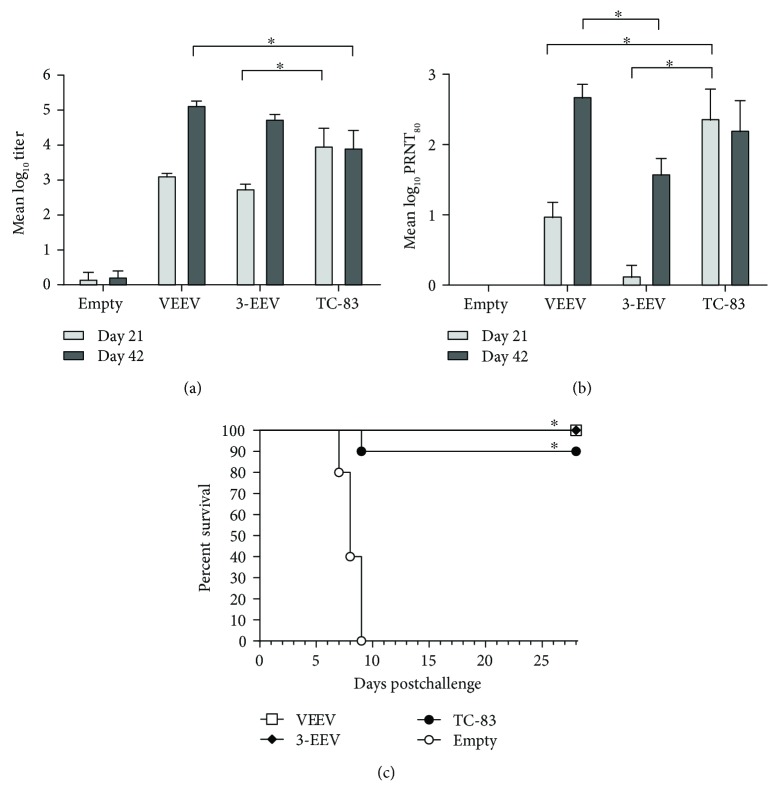
VEEV-specific antibody responses and survival of vaccinated mice. Female BALB/c mice (*n* = 10 per group) were vaccinated on days 0 and 21 with 5 *μ*g of empty vector DNA, 5 *μ*g of the VEEV DNA vaccine, or 5 *μ*g each of the VEEV, WEEV, and EEEV DNA vaccines (3-EEV DNA vaccine) delivered by IM EP or on day 0 with 0.5 ml of the live-attenuated VEEV IND vaccine TC-83 (1 × 10^4^ PFU) delivered by subcutaneous injection. Serum samples obtained on days 21 and 42 were assayed for total IgG anti-VEEV antibodies by ELISA and for VEEV-neutralizing antibodies by PRNT. The group mean log_10_ ELISA (a) and PRNT_80_ (b) titers along with the standard error of the mean (SEM) are shown. ^∗^*p* < 0.05 for comparison of titers between groups. Four weeks after the final vaccination, the mice were challenged with 1 × 10^4^ PFU (~10,000 LD_50_) of VEEV IAB strain Trinidad donkey by the aerosol route. Kaplan-Meier survival curves indicating the percentage of surviving mice at each day of the 28-day postchallenge observation period are shown (c). ^∗^*p* < 0.05 for survival rate and survival curve as compared to negative control group.

Mice vaccinated with either the VEEV DNA or the 3-EEV DNA developed a mean ELISA titer that was significantly above background after a single vaccination (*p* < 0.0001) and that was significantly boosted with a second vaccination (*p* < 0.0001) (Figure 1(a)). In addition, the mean titers of mice vaccinated with the VEEV DNA or the 3-EEV DNA were not significantly different from one another on day 21 (*p* = 0.7702) or 42 (*p* = 0.7328). Although the day 21 mean titer of mice that received TC-83 trended higher than that of mice that received the VEEV DNA vaccine, the difference was not significant (*p* = 0.1258). By day 42, the mean titer of mice that received a second dose of the VEEV DNA was significantly higher than that of mice that received the single dose of TC-83 (*p* = 0.0112). Although the day 21 mean titer of mice vaccinated with the 3-EEV DNA was significantly lower than that of mice vaccinated with TC-83 (*p* < 0.0111), there was no significant difference between the day 42 mean titers of these groups (*p* = 0.1456).

Mice vaccinated with the VEEV DNA developed a mean PRNT_80_ titer that was significantly above background on day 21 (*p* = 0.0260) (Figure 1(b)). In contrast, the day 21 mean titers of mice that received the 3-EEV DNA vaccine were low and not significantly different from those that received the empty vector DNA (*p* = 0.9768). Within groups vaccinated with either the VEEV DNA or 3-EEV DNA, the mean titer was significantly higher on day 42 as compared to that on day 21 (*p* < 0.0001). Although the mean titers of mice that received the VEEV DNA or 3-EEV DNA were not significantly different from one another on day 21 (*p* = 0.0723), the day 42 mean titer of mice that received the VEEV DNA was significantly higher than that of mice that received the 3-EEV DNA (*p* = 0.0106). In addition, although the mean titer of mice vaccinated with TC-83 was significantly higher than that of mice vaccinated with the VEEV DNA (*p* < 0.0007) or the 3-EEV DNA (*p* < 0.0001) on day 21, there was no significant difference between the day 42 mean titer of mice vaccinated with TC-83 as compared to that of mice vaccinated with the VEEV DNA (*p* = 0.5403) or 3-EEV DNA (*p* = 0.2782).

### 3.2. VEEV Aerosol Challenge of Vaccinated Mice

The mice from all groups were challenged on day 49 with 1 × 10^4^ PFU (~10,000 median lethal doses [LD_50_]) of VEEV IAB strain Trinidad donkey by the aerosol route. Negative control mice that received the empty vector DNA all displayed clinical signs of disease including ruffled fur, weight loss, inactivity, hunched posture, ataxia, and hind limb paralysis, and all succumbed to infection or were euthanized in accordance with early endpoint criteria by day 9 postchallenge (Figure 1(c)). In contrast, mice vaccinated with the VEEV DNA or 3-EEV DNA displayed no clinical signs of disease postchallenge and all survived. Consistent with our previous results [32, 38], 90% of mice vaccinated with TC-83 displayed no clinical signs of disease postchallenge and survived, and the single mouse from this group that did not survive the challenge had no detectable VEEV-specific antibody response after vaccination. The survival of the VEEV DNA, 3-EEV DNA, and TC-83 groups was significantly higher than that of the empty vector DNA group with respect to survival rate (*p* < 0.0001) and the survival curve (*p* = 0.0003).

### 3.3. VEEV-Specific Cellular Immune Responses of Vaccinated Mice

**Figure 2:**
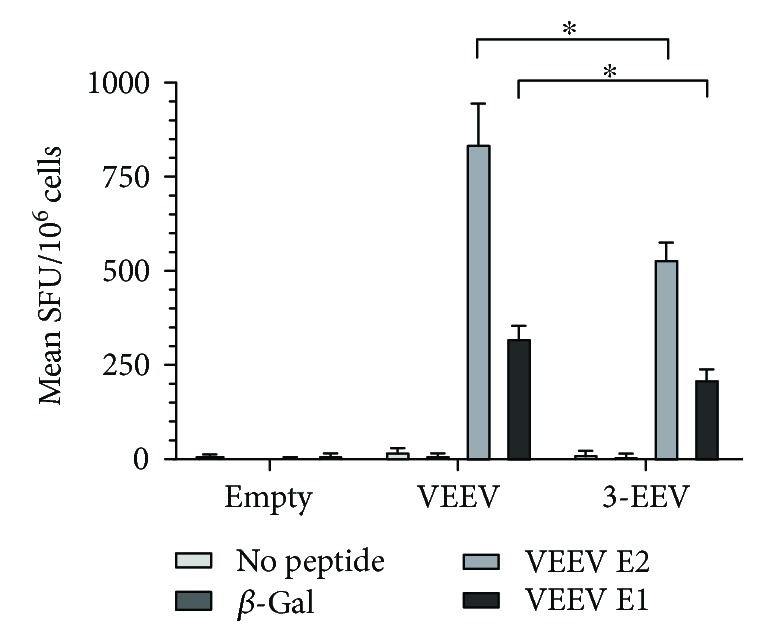
VEEV-specific cellular immune responses of vaccinated mice. Female BALB/c mice (*n* = 6 per group) were vaccinated twice at a 3-week interval with 5 *μ*g of empty vector DNA, 5 *μ*g of the VEEV DNA vaccine, or 5 *μ*g each of the VEEV, WEEV, and EEEV DNA vaccines (3-EEV DNA vaccine) delivered by IM EP. Two weeks after the second vaccination, splenocytes were isolated and restimulated with no peptide, a peptide from the unrelated *β*-galactosidase protein, or pools of overlapping peptides spanning the VEEV IAB E2 or E1 envelope glycoproteins and analyzed by IFN-*γ* ELISpot assay. The mean spot forming units (SFU) per 10^6^ cells along with the SEM are shown for each group. ^∗^*p* < 0.05 for comparison of spot counts between groups.

Previously, we showed that delivery of the optimized VEEV DNA vaccine by IM EP resulted in cellular immune responses directed against the VEEV E2 and E1 proteins as detected by INF*γ*-ELISpot assay [38]. To compare the cellular responses elicited by the VEEV DNA vaccine and the 3-EEV DNA vaccine, female BALB/c mice (*n* = 6 per group) were vaccinated on days 0 and 21 with 5 *μ*g of the empty vector plasmid, 5 *μ*g of the VEEV plasmid, or 5 *μ*g of each of the VEEV, WEEV, and EEEV plasmids (15 *μ*g total) delivered by IM EP. On day 35, splenocytes isolated from the vaccinated mice were restimulated with concanavalin A, no peptide, an irrelevant *β*-galactosidase peptide, or pools of overlapping peptides spanning the VEEV IAB strain Trinidad donkey E2 or E1 envelope glycoproteins and analyzed by IFN-*γ* ELISpot. After restimulation with concanavalin A, splenocytes from mice from all groups produced spots that were too numerous to count (data not shown). Splenocytes restimulated with no peptide (*p* ≥ 0.5964) or with the *β*-galactosidase peptide (*p* ≥ 0.1515) failed to produce significant responses in this assay. After restimulation with the VEEV E2 or E1 peptide pools, splenocytes obtained from mice vaccinated with the VEEV DNA (*p* < 0.0001) or 3-EEV DNA (*p* ≤ 0.0010) produced mean IFN-*γ* responses that were significantly above background (Figure 2). However, the mean IFN-*γ* responses of mice receiving the VEEV DNA were significantly higher than those of mice receiving the 3-EEV DNA against the E2 (*p* = 0.0218) and E1 (*p* = 0.0180) peptide pools. Consistent with our previous results, the mean IFN-*γ* responses of splenocytes restimulated with the E2 peptides were significantly higher than those restimulated with the E1 peptides for both the VEEV DNA (*p* = 0.0142) and 3-EEV DNA (*p* = 0.0010) groups.

### 3.4. WEEV-Specific Antibody Responses of Vaccinated Mice

To perform a comparative evaluation of the immunogenicity and protective efficacy of the individual optimized WEEV DNA and 3-EEV DNA vaccines, female BALB/c mice (*n* = 10 per group) were vaccinated on days 0 and 21 with 5 *μ*g of the WEEV plasmid or with 5 *μ*g of each of the VEEV, WEEV, and EEEV plasmids (15 *μ*g total) by IM EP. Negative control mice (*n* = 10) were vaccinated on days 0 and 21 with 5 *μ*g of the empty vector plasmid by IM EP. To allow comparison to the formalin-inactivated WEEV IND vaccine, mice (*n* = 10) were vaccinated on days 0 and 21 with the human dose of 0.5 ml of this vaccine by subcutaneous injection. Serum samples obtained on days 21 and 42 were assayed for total IgG anti-WEEV antibodies by ELISA and for WEEV-neutralizing antibodies by PRNT.

**Figure 3:**
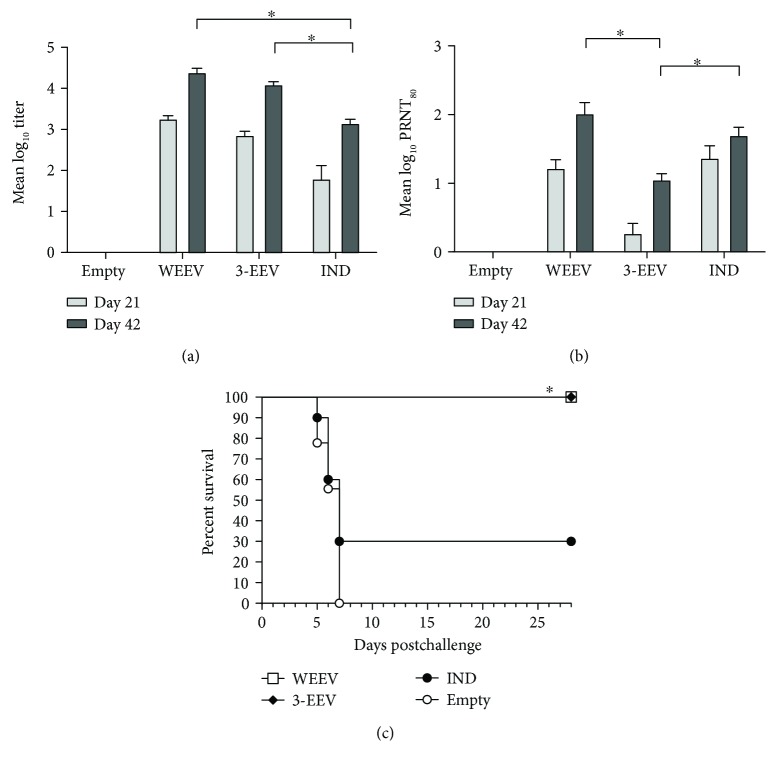
WEEV-specific antibody responses and survival of vaccinated mice. Female BALB/c mice (*n* = 10 per group) were vaccinated on days 0 and 21 with 5 *μ*g of empty vector DNA, 5 *μ*g of the WEEV DNA vaccine, or 5 *μ*g each of the VEEV, WEEV, and EEEV DNA vaccines (3-EEV DNA vaccine) delivered by IM EP or 0.5 ml of the formalin-inactivated WEEV IND vaccine delivered by subcutaneous injection. Serum samples obtained on days 21 and 42 were assayed for total IgG anti-WEEV antibodies by ELISA and for WEEV-neutralizing antibodies by PRNT. The group mean log_10_ ELISA (a) and PRNT_80_ (b) titers along with the SEM are shown. ^∗^*p* < 0.05 for comparison of titers between groups. Four weeks after the final vaccination, the mice were challenged with 2 × 10^4^ PFU (~500 LD_50_) of WEEV strain CBA87 by the aerosol route. Kaplan-Meier survival curves indicating the percentage of surviving mice at each day of the 28-day postchallenge observation period are shown (c). ^∗^*p* < 0.05 for survival rate as compared to negative control group.

Mice that received the WEEV DNA vaccine, 3-EEV DNA vaccine, or WEEV IND vaccine developed mean ELISA titers that were significantly above background after a single vaccination (*p* < 0.0001) and that were significantly boosted with a second vaccination (*p* ≤ 0.0007) (Figure 3(a)). The mean titers of mice vaccinated with the WEEV DNA or 3-EEV DNA were not significantly different from one another on day 21 (*p* = 0.1435) or 42 (*p* = 0.4116). In addition, the mean titers of mice vaccinated with the WEEV DNA or 3-EEV DNA were statistically higher than that of mice receiving the WEEV IND vaccine at day 21 (*p* ≤ 0.0004) and 42 (*p* < 0.0001).

Mice vaccinated with the WEEV DNA developed a mean PRNT_80_ titer that was significantly above background after a single vaccination (*p* < 0.0001) and that was significantly boosted with a second vaccination (*p* = 0.0011) (Figure 3(b)). In contrast, although mice that received a single vaccination with the 3-EEV DNA did not develop a mean titer that was significantly above background (*p* = 0.4304), the mean titer of these mice was significantly boosted (*p* = 0.0004) and was significantly above background after a second vaccination (*p* < 0.0001). Although the mean titer of mice that received the WEEV IND vaccine was significantly above background after a single vaccination (*p* < 0.0001), the mean titer was not significantly boosted with a second vaccination (*p* = 0.0596). In comparing the mean titers between groups, the titers of mice that received the WEEV DNA or WEEV IND vaccine were not significantly different on day 21 (*p* = 0.8361) or 42 (*p* = 0.1557). However, the mean titer of mice that received the 3-EEV DNA vaccine was significantly lower than those of mice that received the WEEV DNA or WEEV IND vaccine at day 21 (*p* < 0.0001) and 42 (*p* ≤ 0.0004).

### 3.5. WEEV Aerosol Challenge of Vaccinated Mice

The mice from all groups were challenged on day 49 with 2 × 10^4^ PFU (~500 LD_50_) of WEEV strain CBA87 by the aerosol route. Negative control mice that received the empty vector DNA all displayed clinical signs of disease including ruffled fur, weight loss, inactivity, hunched posture, ataxia, and hind limb paralysis, and all succumbed to infection or were euthanized in accordance with early endpoint criteria by day 7 postchallenge (Figure 3(c)). In contrast, mice vaccinated with the WEEV DNA or 3-EEV DNA displayed no clinical signs of disease postchallenge and all survived. Consistent with our previous unpublished results, only 30% of the mice that received the WEEV IND vaccine survived the challenge. The survival of the WEEV DNA and 3-EEV DNA groups was significantly higher than that of the WEEV IND group with respect to the survival rate (*p* = 0.0030) and survival curve (*p* = 0.0056). In addition, the survival of the empty vector DNA and WEEV IND groups were not significantly different with respect to the survival rate (*p* = 0.2101), mean time-to-death (*p* = 0.8420), and survival curve (*p* = 0.2856).

### 3.6. EEEV-Specific Antibody Responses of Vaccinated Mice

We also completed a comparative evaluation of the immunogenicity and protective efficacy of the individual optimized EEEV DNA and 3-EEV DNA vaccines delivered by IM EP in mice. In our unpublished studies, it has proven difficult to elicit protective immunity in mice against EEEV aerosol challenge. Consequently, for this study, we vaccinated female BALB/c mice (*n* = 10 per group) three times, instead of twice, on days 0, 21, and 42 with 5 *μ*g of the EEEV plasmid or with 5 *μ*g of each of the VEEV, WEEV, and EEEV plasmids (15 *μ*g total) by IM EP. Negative control mice (*n* = 10) were vaccinated on days 0, 21, and 42 with 5 *μ*g of the empty vector plasmid by IM EP. To allow comparison to the formalin-inactivated EEEV IND vaccine, mice (*n* = 10) were vaccinated on days 0, 21, and 42 with the human dose of 0.5 ml of this vaccine by subcutaneous injection. Serum samples obtained on days 21, 42, and 63 were assayed for total IgG anti-EEEV antibodies by ELISA and for EEEV-neutralizing antibodies by PRNT.

**Figure 4:**
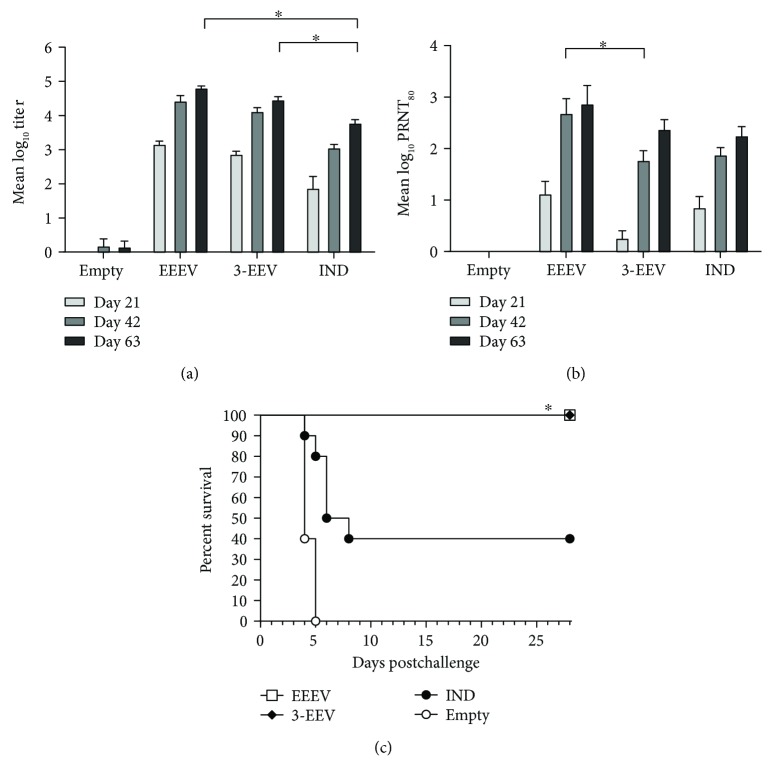
EEEV-specific antibody responses and survival of vaccinated mice. Female BALB/c mice (*n* = 10 per group) were vaccinated on days 0, 21, and 42 with 5 *μ*g of empty vector DNA, 5 *μ*g of the EEEV DNA vaccine, or 5 *μ*g each of the VEEV, WEEV, and EEEV DNA vaccines (3-EEV DNA vaccine) delivered by IM EP or 0.5 ml of the formalin-inactivated EEEV IND vaccine delivered by subcutaneous injection. Serum samples obtained on days 21, 42, and 63 were assayed for total IgG anti-EEEV antibodies by ELISA and for EEEV-neutralizing antibodies by PRNT. The group mean log_10_ ELISA (a) and PRNT_80_ (b) titers along with the SEM are shown. ^∗^*p* < 0.05 for comparison of titers between groups. Four weeks after the final vaccination, the mice were challenged with 1 × 10^5^ PFU (~3000 LD_50_) of EEEV strain FL91-4679 by the aerosol route. Kaplan-Meier survival curves indicating the percentage of surviving mice at each day of the 28-day postchallenge observation period are shown (c). ^∗^*p* < 0.05 for survival rate as compared to negative control group.

Mice that received the EEEV DNA vaccine, 3-EEV DNA vaccine, or EEEV IND vaccine developed mean ELISA titers that were significantly above background after a single vaccination (*p* < 0.0001) and that were significantly boosted with a second vaccination (*p* ≤ 0.0040) (Figure 4(a)). While the mean titer of mice vaccinated with the EEEV DNA was not significantly boosted with a third vaccination (*p* = 0.0508), those of mice that received the 3-EEV DNA or EEEV IND vaccine were significantly higher on day 63 as compared to day 42 (*p* ≤ 0.0432). In comparing the mean titers between groups, the titers of mice vaccinated with the EEEV DNA or 3-EEV DNA were not significantly different from one another on day 21 (*p* = 0.9280), 42 (*p* = 0.7396), or 63 (*p* = 0.1267). In addition, the mean titers of mice vaccinated with the EEEV DNA or 3-EEV DNA were significantly higher than those of mice receiving the EEEV IND vaccine on day 21 (*p* ≤ 0.0021), 42 (*p* < 0.0001), and 63 (*p* < 0.0001).

Mice that received the EEEV DNA vaccine developed a mean PRNT_80_ titer that was significantly above background after a single vaccination (*p* = 0.0030) and significantly boosted with a second vaccination (*p* < 0.0001), but not significantly boosted with a third vaccination (*p* = 0.4473) (Figure 4(b)). Although the mean titers of mice that received the 3-EEV DNA or EEEV IND vaccine were not significantly above background after a single vaccination (*p* ≥ 0.0538), they were significantly boosted (*p* ≤ 0.0002) and significantly above background after a second vaccination (*p* < 0.0001). The mean titers of the 3-EEV DNA or EEEV IND vaccine groups were also significantly boosted with a third vaccination (*p* ≤ 0.0310). In comparing the mean titers between groups, the titers of mice vaccinated with the EEEV DNA or 3-EEV DNA were not significantly different from one another on day 21 (*p* = 0.0533) and 63 (*p* = 0.5463), while the day 42 titer of the EEEV DNA group was significantly higher than that of the 3-EEV DNA group (*p* = 0.0346). In addition, the mean titer of mice that received the EEEV IND vaccine was not significantly different from those of mice vaccinated with the EEEV DNA or 3-EEV DNA at day 21 (*p* ≥ 0.4041), 42 (*p* ≥ 0.0927), or 63 (*p* ≥ 0.2960).

### 3.7. EEEV Aerosol Challenge of Vaccinated Mice

The mice from all groups were challenged on day 70 with 1 × 10^5^ PFU (~3000 LD_50_) of EEEV strain FL91-4679 by the aerosol route. Negative control mice that received the empty vector DNA all displayed clinical signs of disease including ruffled fur, weight loss, inactivity, hunched posture, ataxia, and hind limb paralysis, and all succumbed to infection or were euthanized in accordance with early endpoint criteria by day 5 postchallenge (Figure 4(c)). In contrast, mice vaccinated with the EEEV DNA or 3-EEV DNA displayed no clinical signs of disease postchallenge and all survived. Consistent with our previous unpublished results, only 40% of the mice that received the EEEV IND vaccine survived the challenge. The survival rates of the EEEV DNA and 3-EEV DNA groups were significantly higher than that of the EEEV IND group (*p* = 0.0329). Although the survival rates of mice receiving the EEEV IND group and the empty vector DNA group were not statistically different (*p* = 0.3025), the survival of the EEEV IND group was significantly enhanced relative to that of the empty vector DNA group with respect to the mean time-to-death (*p* = 0.0452) and the survival curve (*p* = 0.0066). Of note, mice that received only two vaccinations with the EEEV DNA vaccine were also completely protected from challenge (data not shown).

### 3.8. Virus-Specific Antibody Responses of Vaccinated Rabbits

To perform a comparative evaluation of the immunogenicity of the individual optimized VEEV, WEEV, and EEEV DNA vaccines and the 3-EEV DNA vaccine in an additional animal model that permits administration of higher DNA doses that are more similar to those expected to be delivered to humans and is better suited to assessment of antibody durability, we also completed a study in rabbits. New Zealand White rabbits (*n* = 5 per group) were vaccinated on days 0, 28, and 230 with 0.5 mg of the VEEV, WEEV, or EEEV plasmid or with 0.5 mg each of the VEEV, WEEV, and EEEV DNA plasmids (1.5 mg total) delivered by IM EP. Serum samples obtained on days 27, 42, 230, 266, and 349 were assayed for neutralizing antibodies against VEEV, WEEV, or EEEV by PRNT.

**Figure 5:**
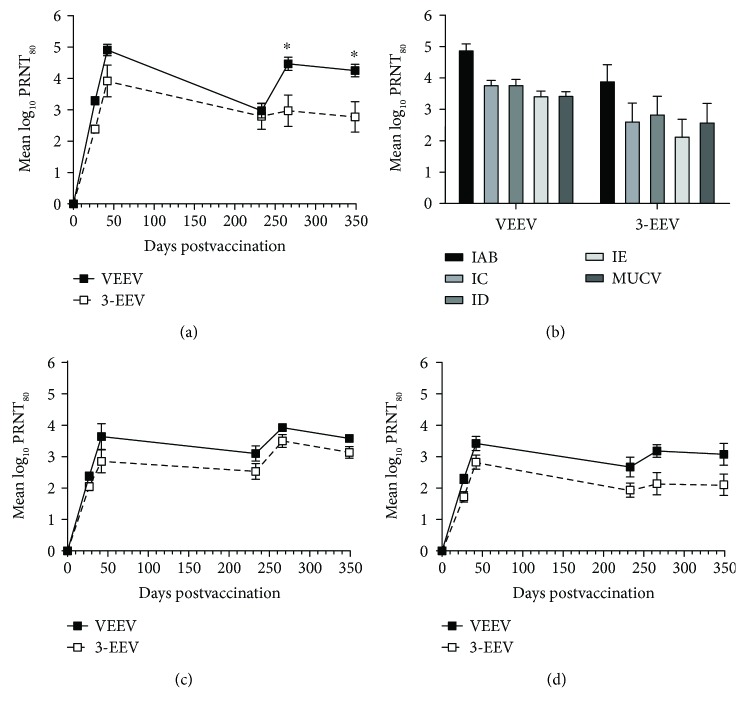
Virus-neutralizing antibody responses of vaccinated rabbits. New Zealand White rabbits (*n* = 5 per group) were vaccinated on days 0, 28, and 230 with 0.5 mg of the VEEV, WEEV, or EEEV DNA vaccine or with 0.5 mg each of the VEEV, WEEV, and EEEV DNA vaccines (3-EEV DNA vaccine) delivered by IM EP. Serum samples obtained on days 27, 42, 230, 266, and 349 were assayed for neutralizing antibodies against VEEV IAB (a), WEEV (c), or EEEV (d) by PRNT. The day 42 serum samples from rabbits vaccinated with the VEEV DNA or 3-EEV DNA were also assayed for neutralizing activity against heterologous VEEV subtypes IC, ID, and IE and MUCV (b) by PRNT. The group mean log_10_ PRNT_80_ titers along with the SEM are shown. ^∗^*p* < 0.05 for comparison of titers between groups.

Rabbits that received the VEEV DNA vaccine or 3-EEV DNA vaccine developed mean PRNT_80_ titers against VEEV that were significantly above background after a single vaccination (*p* < 0.0001) and significantly boosted with a second vaccination (*p* < 0.0001) (Figure 5(a)). While the day 230 mean titer of rabbits vaccinated with the VEEV DNA was significantly lower than that on day 42 (*p* = 0.0004), there was no significant difference in the day 42 and day 230 mean titers for rabbits vaccinated with the 3-EEV DNA (*p* = 0.2827). The mean titer of rabbits that received the VEEV DNA was also significantly boosted with the long-range boosting vaccination performed on day 230 (*p* = 0.0133). Although the long-range boosting vaccination increased the mean log_10_ titer of rabbits that received the 3-EEV DNA from 2.80 on day 230 to 2.97 on day 266, this increase was not statistically significant (*p* > 0.9999). In addition, there was no significant difference in the day 266 and day 349 mean titers of rabbits vaccinated with the VEEV DNA or 3-EEV DNA within these groups (*p* > 0.9999). In comparing the mean titers between groups, there was no significant difference in the titers of rabbits vaccinated with the VEEV DNA or 3-EEV DNA at day 27 (*p* = 0.523), 42 (*p* = 0.3935), and 230 (*p* > 0.9999). However, after the long-range boosting vaccination, the mean titers of rabbits that received the VEEV DNA vaccine were significantly higher than those of rabbits that received the 3-EEV DNA vaccine at day 266 (*p* = 0.0252) and 349 (*p* = 0.0464).

To assess the potential for the subtype IAB-based VEEV DNA vaccine to provide protection against heterologous VEEV strains, we measured the neutralizing activity of the day 42 samples from rabbits vaccinated with the VEEV DNA or 3-EEV DNA against VEEV subtypes IC, ID, and IE and MUCV (formerly VEEV IIIA). Within groups receiving the VEEV DNA or 3-EEV DNA, there was no significant difference in the mean PRNT_80_ titers against VEEV subtypes IAB, IC, ID, or IE or MUCV (*p* ≥ 0.0587) (Figure 5(b)). In comparing the mean titers between groups, there was no significant difference in the titers of rabbits vaccinated with the VEEV DNA or 3-EEV DNA against VEEV subtypes IAB, IC, ID, or IE or MUCV (*p* ≥ 0.2802).

Rabbits that received the WEEV DNA vaccine or 3-EEV DNA vaccine developed mean PRNT_80_ titers against WEEV that were significantly above background after a single vaccination (*p* < 0.0001) (Figure 5(c)). Although the mean titer of rabbits vaccinated with the WEEV DNA was significantly boosted with a second vaccination (*p* = 0.005), there was no significant difference in the day 27 and day 42 mean titers of rabbits vaccinated with the 3-EEV DNA (*p* = 0.394). There was also no significant difference in the day 42 and day 230 mean titers for rabbits vaccinated with the WEEV DNA (*p* = 0.7824) or 3-EEV DNA (*p* = 0.9976). Although the long-range boosting vaccination increased the mean log_10_ titer from 3.10 on day 230 to 3.93 on day 266 for rabbits receiving the WEEV DNA and from 2.53 on day 230 to 3.50 on day 266 for rabbits receiving the 3-EEV DNA, these increases were not statistically significant (*p* ≥ 0.1551). In addition, there was no significant difference in the day 266 and day 349 mean titers of rabbits vaccinated with the WEEV DNA or 3-EEV DNA within these groups (*p* ≥ 0.9917). In comparing the mean titers between groups, there was no significant difference in the titers of rabbits vaccinated with the WEEV DNA or 3-EEV DNA at any of the time points (*p* ≥ 0.3404).

Rabbits that received the EEEV DNA vaccine or 3-EEV DNA vaccine developed mean PRNT_80_ titers against EEEV that were significantly above background after a single vaccination (*p* ≤ 0.0013) (Figure 5(d)). Although the mean titer of rabbits vaccinated with the EEEV DNA was significantly boosted with a second vaccination (*p* = 0.048), there was no significant difference in the mean titers at day 27 and day 42 for rabbits vaccinated with the 3-EEV DNA (*p* = 0.135). There was also no significant difference in the day 42 and day 230 mean titers for rabbits vaccinated with the EEEV DNA (*p* = 0.4883) or 3-EEV DNA (*p* = 0.3987). Although the long-range boosting vaccination increased the mean log_10_ titer from 2.67 on day 230 to 3.18 on day 266 for rabbits receiving the EEEV DNA and from 1.94 on day 230 to 2.14 on day 266 for rabbits receiving the 3-EEV DNA, these increases were not statistically significant (*p* ≥ 0.9108). In addition, there was no significant difference in the day 266 and day 349 mean titers of rabbits vaccinated with the EEEV DNA or 3-EEV DNA within these groups (*p* > 0.9999). In comparing the mean titers between groups, there was no significant difference in the titers of rabbits vaccinated with the EEEV DNA or 3-EEV DNA at any of the time points (*p* ≥ 0.1383).

## 4. Discussion

The results of our previous studies demonstrated that a strategy that encompassed optimization of the construct for increased antigen expression and EP-based delivery successfully improved the immunogenicity and protective efficacy of a VEEV DNA vaccine [38]. Consistent with those results, mice that received two doses of the optimized VEEV DNA vaccine delivered by IM EP in the present studies developed robust virus-specific total IgG and virus-neutralizing antibody responses. Comparison against mice that received a single vaccination with a human dose of the live-attenuated VEEV IND vaccine TC-83 revealed that the virus-specific total IgG titers elicited by the VEEV DNA vaccine were significantly higher than those observed for TC-83, while the virus-neutralizing antibody responses were similar between these two vaccination regimens. Also consistent with our previous results, mice that received the VEEV DNA vaccine were completely protected against lethal VEEV aerosol challenge, whereas 90% of mice receiving TC-83 were protected. In a similar manner, mice that received the optimized WEEV or EEEV DNA vaccine delivered by IM EP developed robust virus-specific total IgG and virus-neutralizing antibody responses. Comparison against mice that received the same number of vaccinations with human doses of the formalin-inactivated WEEV or EEEV IND vaccine revealed that the virus-specific total IgG titers elicited by the WEEV or EEEV DNA vaccine were significantly higher than those observed for the respective WEEV or EEEV IND vaccine, while the virus-neutralizing antibody responses were similar between these vaccination regimens. Mice that received the WEEV or EEEV DNA vaccine were also completely protected from lethal homologous WEEV or EEEV aerosol challenge and exhibited significantly higher survival rates than were mice that received the WEEV or EEEV IND vaccine, which only protected 30% and 40% of vaccinated mice, respectively. These results demonstrate that this vaccination strategy was also successful in developing protective DNA vaccines for WEEV and EEEV that provide significantly increased protection against lethal viral aerosol challenge in mice compared to the formalin-inactivated IND vaccines.

In the present studies, we also evaluated whether the optimized VEEV, WEEV, and EEEV DNA vaccines could elicit immune responses adequate for protection when administered in a multiagent formulation. While the virus-specific total IgG antibody titers of mice that received the individual VEEV, WEEV, or EEEV DNA vaccine were similar to those of mice that received the 3-EEV DNA vaccine, the virus-neutralizing antibody titers were significantly lower in mice that received the 3-EEV DNA vaccine compared to those that received the individual VEEV or WEEV DNA vaccine. Therefore, it is possible that some level of interference occurs when the three different but related vaccine antigens are expressed in the same target tissue. However, this may also be a function of competition for antigen production based on the larger amount of DNA delivered to the same tissue for the 3-EEV DNA as compared to the individual DNA vaccines. Despite these observed differences, all of the mice that received the 3-EEV DNA vaccine had detectable neutralizing antibody responses against VEEV, WEEV, and EEEV and were completely protected against lethal VEEV, WEEV, and EEEV aerosol challenge. As observed for the individual VEEV, WEEV, and EEEV DNA vaccines, the 3-EEV DNA vaccine also provided similar levels of protection against lethal VEEV aerosol challenge as compared to TC-83 and significantly increased protection against lethal WEEV and EEEV aerosol challenge as compared to the formalin-inactivated WEEV and EEEV IND vaccines in mice. Furthermore, there was no significant difference in the neutralizing antibody responses against VEEV, WEEV, and EEEV elicited by the individual DNA vaccines or 3-EEV DNA vaccine after the initial vaccination series in rabbits. These results provide important preliminary evidence to support the potential use of the 3-EEV DNA as a single multiagent vaccine formulation capable of eliciting protective immunity against VEEV, WEEV, and EEEV.

Of note, there have been previous published reports on the evaluation of WEEV DNA vaccines in mice. In one report, a DNA vaccine expressing the structural proteins (C-E3-E2-6K-E1) of WEEV strain 71V-1658 from the wild-type genes administered in four 5 *μ*g doses by PMED provided complete protection against homologous intranasal challenge with 1.5 × 10^3^ PFU (25 LD_50_) of virus [46]. However, this vaccine provided only partial protection against similar challenges with the heterologous WEEV strains CBA87 and Fleming. Although cell-mediated immune responses against the E2 and E1 antigens were elicited by this DNA vaccine as measured by lymphocyte proliferation assays, no virus-specific antibody responses were detected by ELISA. In a subsequent report by this group, DNA vaccines expressing the C-E3-E2-6K-E1, E3-E2-6K-E1, or 6K-E1 proteins of WEEV strain 71V-1658 from the wild-type genes administered in three 2 *μ*g doses by PMED provided complete protection against homologous intranasal challenge with the same 1.5 × 10^3^ PFU (25 LD_50_) dose of virus, while a DNA vaccine expressing the E3-E2 proteins did not provide any protection [47]. Although the DNA vaccines expressing the C-E3-E2-6K-E1, E3-E2-6K-E1, or 6K-E1 proteins provided significant protection against a similar challenge with the CBA87 strain, only the DNA vaccines expressing the C-E3-E2-6K-E1 and E3-E2-6K-E1 proteins provided significant protection against the Fleming strain. In addition, the DNA vaccine expressing the E3-E2-6K-E1 proteins provided better protection against this strain than the DNA vaccine expressing C-E3-E2-6K-E1. In our studies, we showed that two administrations of a 5 *μ*g dose of a DNA vaccine expressing E3-E2-6K-E1 proteins of WEEV CBA87 from codon-optimized genes delivered by IM EP provided complete protection against aerosol challenge with 2 × 10^4^ PFU (~500 LD_50_) of homologous virus. Taken together, the described results of the studies previously performed by others and of those reported here support the use of E3-E2-6K-E1 as the most appropriate target antigens for a successful DNA vaccination strategy against encephalitic alphaviruses. However, our results indicate that it is likely that codon optimization of the structural genes in the construct along with the efficiency of EP-based delivery contributed to the ability of the DNA vaccine evaluated here to protect against the higher challenge dose with fewer DNA administrations. Because no immunogenicity results were provided in the report by Gauci et al., it is not possible to make an indirect comparison of the immunogenicity of the previously tested WEEV DNA vaccines with that of the one we evaluated here.

It should also be noted that evaluation of individual and combined VEEV, WEEV, and EEEV virus replicon particle (VRP) vaccines in mice and NHPs has also been recently reported. In these experiments, the individual VRP vaccines delivered twice at a dose of 1 × 10^7^ infectious units elicited strong and durable virus-specific antibody responses in mice as measured by ELISA and PRNT and provided complete protection against homologous lethal VEEV, WEEV, and EEEV aerosol challenges [48]. The VEEV VRP vaccine based on the IAB strain was also shown to elicit durable protective immunity in mice against lethal aerosol challenge with the heterologous VEEV strain IE and MUCV. In the murine studies, there were also no significant differences in the antibody or protection levels when the VRP vaccines were administered in combination. While the individual VEEV and EEEV and combination VRP vaccines protected NHPs against homologous VEEV and EEEV aerosol challenge, the protection elicited by the WEEV or combination VRP vaccines against WEEV aerosol challenge was not significantly different from that of mock-vaccinated controls. The DNA vaccines evaluated in our studies reported here compare favorably to the VRP vaccines in that complete protection in mice against the same challenge doses of aerosolized VEEV, WEEV, and EEEV was also afforded by the individual and 3-EEV DNA vaccines. Although we did not directly assess the duration of protective immunity elicited by the individual and 3-EEV DNA vaccines in the mouse studies reported here, our results in rabbits demonstrated that virus-neutralizing antibody titers elicited by these vaccines remained significantly above background out to 349 days after the initial vaccination. We also showed that sera from rabbits that received the subtype IAB-based VEEV DNA vaccine administered individually or in the 3-EEV DNA formulation had high levels of neutralizing activity against heterologous VEEV subtypes IC, ID, and IE and MUCV. While these results are indicative of the potential for the individual and 3-EEV DNA vaccines to elicit durable protective immunity and for the VEEV DNA and 3-EEV DNA vaccines to protect against heterologous VEEV subtypes, we are currently completing studies to directly evaluate these possibilities. We are also currently completing studies to evaluate the immunogenicity and protective efficacy of the individual and 3-EEV DNA vaccines delivered by EP against VEEV, WEEV, and EEEV aerosol challenge in NHPs. The results of these studies will be important for further comparisons to the VRP and other next-generation alphavirus vaccine candidates.

The most widely accepted correlate of protection against the encephalitic alphaviruses is neutralizing antibodies directed against the envelope glycoproteins [49–53]. However, neutralizing antibody titers are not always significantly associated with protection against encephalitic alphavirus challenge by the aerosol route [54–56]. In the studies reported here, the VEEV, WEEV, and EEEV DNA vaccines elicited robust virus-specific antibody responses, to include detectable levels of virus-neutralizing antibodies, when delivered individually or in a multiagent formulation. Although we observed that mice that received the individual WEEV DNA or WEEV IND vaccine had similar virus-neutralizing antibody titers, those that received the WEEV DNA vaccine were completely protected from WEEV aerosol challenge and had significantly improved protection as compared to mice that received the WEEV IND vaccine. More strikingly, mice that received the 3-EEV DNA vaccine were also completely protected from WEEV aerosol challenge and had significantly improved protection as compared to mice that received the WEEV IND vaccine despite having significantly lower virus-neutralizing antibody titers. Similarly, although mice that received the individual EEEV DNA, the 3-EEV DNA, or the EEEV IND vaccine had similar virus-neutralizing antibody titers, those that received the EEEV DNA or 3-EEV DNA vaccine were completely protected from EEEV aerosol challenge and had significantly improved protection as compared to mice that received the EEEV IND vaccine. The ability of nonneutralizing antibodies to also mediate protection against encephalitis caused by alphaviruses has been previously documented [57, 58]. Therefore, it is likely that nonneutralizing antibody responses elicited by the individual VEEV, WEEV, and EEEV DNA vaccines and 3-EEV DNA vaccine also contributed to the protection levels observed in the present studies. This is supported by our observation that mice that received the individual WEEV, individual EEEV, or 3-EEV DNA vaccine had significantly higher virus-specific total IgG antibody titers than mice receiving the respective IND vaccine. Roles for mucosal antibody responses and antibody-dependent cellular cytotoxicity in protection against aerosol VEEV challenge in mice have also been documented [59–61]. Therefore, we are currently performing a more thorough characterization of the antibody responses elicited by the individual VEEV, WEEV, and EEEV DNA vaccines and 3-EEV DNA vaccine to further elucidate the contributing role of these responses in the protection observed for these vaccines against VEEV, WEEV, and EEEV aerosol challenge.

Although cytotoxic T cell activity was not observed in previous studies with TC-83, more recent studies have also demonstrated an importance for certain populations of T cells in protection against lethal encephalitis caused by VEEV in mice [62–65]. In our previous studies, we demonstrated that the optimized VEEV DNA vaccine delivered by IM EP elicited significant cell-mediated immune responses against the VEEV E2 and E1 glycoproteins as measured by IFN-*γ* ELISpot assay [38]. The ELISpot assay results obtained for the individual VEEV DNA vaccine in our current studies were consistent with those previous results. Although the 3-EEV DNA vaccine elicited significantly lower responses against the VEEV E2 and E1 proteins as compared to the individual VEEV DNA vaccine in this assay, they remained at significant levels. Therefore, it is possible that cell-mediated immune responses elicited by the 3-EEV DNA vaccine also contributed to the protection against VEEV aerosol challenge observed here. IFN-*γ* ELISpot assays required to directly measure cell-mediated immune responses against WEEV and EEEV are currently under development in our laboratory, and the results from these assays will be helpful in determining the potential for virus-specific cell-mediated immune responses elicited by the individual WEEV, individual EEEV, and 3-EEV DNA vaccines to contribute to the protection observed for these vaccines against WEEV and EEEV aerosol challenge.

## 5. Conclusions

Taken together, the results of our studies described here clearly demonstrate that the individual VEEV, WEEV, and EEEV DNA vaccines and 3-EEV DNA vaccine delivered by IM EP are capable of eliciting robust and protective immune responses against the encephalitic alphaviruses with relatively low DNA doses and with few vaccinations. To our knowledge, this is the first report of a single nucleic acid-based multiagent vaccine formulation that can provide complete protection against VEEV, WEEV, and EEEV aerosol challenge in mice. Consequently, these DNA vaccines appear to represent a viable next-generation alternative to the current alphavirus IND vaccines. The DNA vaccine platform used here also avoids issues with manufacturing, boosting potential, stability, and safety that can be problematic for other approaches to develop next-generation vaccines. In addition, the results from our completed Phase 1 clinical trial demonstrated the safety, tolerability, and immunogenicity of the VEEV DNA vaccine candidate delivered by IM or ID EP in humans. Therefore, we are currently completing studies to evaluate and compare the immunogenicity and protective efficacy of the individual VEEV, WEEV, and EEEV and 3-EEV DNA vaccines delivered by IM or ID EP in NHPs. Should protective efficacy be successfully demonstrated in these studies, then the individual EEEV, individual WEEV, and 3-EEV DNA vaccines will also be well poised for clinical evaluation.

## Data Availability

The data used to support the findings of these studies were generated under funding from the Joint Science and Technology Office for Chemical and Biological Defense of the Defense Threat and Reduction Agency to USAMRIID and Ichor Medical Systems, and so cannot be made freely available. Access to these data will be considered by the corresponding author upon request, with permission of the Joint Science and Technology Office for Chemical and Biological Defense of the Defense Threat and Reduction Agency, USAMRIID, and Ichor Medical Systems.
